# PFEA: a VLM-based high-level natural language planning and feedback embodied agent for human-centered AI

**DOI:** 10.3389/frobt.2026.1905578

**Published:** 2026-08-06

**Authors:** Wenbin Ding, Jun Chen, Mingjia Chen, Fei Xie, Jun Wang, Simon X. Yang, Philip Dames

**Affiliations:** 1 School of Electrical and Automation Engineering, Nanjing Normal University, Nanjing, Jiangsu, China; 2 Wenzhou Key Laboratory of AI Agents for Agriculture, Wenzhou, Zhejiang, China; 3 Wenzhou Vocational College of Science and Technology, Wenzhou, Zhejiang, China; 4 Key Laboratory of Knowledge Engineering with Big Data (Hefei University of Technology), Ministry of Education, Hefei, Anhui, China; 5 Advanced Robotics and Intelligent Systems Laboratory, College of Engineering, University of Guelph, Guelph, ON, Canada; 6 Department of Mechanical Engineering, Temple University, Philadelphia, PA, United States

**Keywords:** closed-loop planning, feedback mechanism, human-centered embodied agent, robotic task execution, vision-language models, zero-shot manipulation

## Abstract

The rapid advancement of Large Language Models (LLMs) has led to significant progress in Artificial Intelligence (AI), ushering in a new era of human-centered AI (HAI). Intelligent agents powered by LLMs provide new opportunities for realizing HAI. However, existing LLM-based embodied agents often lack online planning capabilities and may generate actions involving objects that are not present in the current environment. In this paper, we propose a closed-loop framework for planning and evaluation of a vision-language model-based robotic manipulation agent operating in tabletop object rearrangement and manipulation tasks. These tasks require a robot to interpret high-level natural language commands and perform multi-step actions such as stacking objects, sorting objects by category or attribute, placing objects into target containers, and organizing mixed desktop scenes. The proposed architecture consists of a human–robot speech interaction module, a vision–language agent module (including a planner, translator, and evaluator), and an action execution module. Within this framework, the vision–language planner decomposes high-level instructions into executable task steps via Chain-of-Objects Reasoning, enabling a deeper understanding of the visual environment, including spatial relationships and object attributes. Meanwhile, the task evaluator monitors execution outcomes and provides result-oriented feedback, allowing the system to replan when errors occur. Experimental results show that, compared with baseline methods, the proposed agent improves the average task success rate by approximately 20% in both simulation and real-world environments, significantly enhancing its ability to execute complex natural language instructions. This work demonstrates the potential of closed-loop vision-language planning for human-centered robotic manipulation. Code: https://github.com/subforpaper/PFEA.

## 1 Introduction

The emergence of Human-centered Artificial Intelligence (HAI) (Capel and Brereton, 2023) has significantly reshaped the field of intelligent robotic systems. In HAI-driven environments, robotic systems must move beyond rigid preprogrammed behaviors and instead enable natural human interaction, environment-aware decision-making, and adaptive task execution. These requirements impose new technical challenges on robotic systems, particularly in enabling robots to understand high-level human intentions and translate them into executable actions in real-world environments.

A key requirement of human-centered robotic systems is the ability to interpret high-level natural language instructions and execute them in dynamic physical environments. In this work, we focus on tabletop object rearrangement and manipulation tasks, including object stacking, attribute-based sorting, object-to-container placement, tool packing, and mixed desktop organization. Instead of relying on predefined workflows, these human-centered tasks are often loosely specified and require robots to reason about objects, spatial relationships, and task goals based on contextual information. Therefore, these robotic systems must be capable of 1) grounding natural language instructions in visual environments, 2) decomposing high-level goals into executable action sequences, and 3) adapting execution strategies based on feedback from the environment.

Large Language Models (LLMs) (Vaswani et al., 2017; Brown et al., 2020) possess extensive semantic knowledge and reasoning capabilities, which enable them to understand complex natural language instructions and perform high-level planning via few-shot prompting. Several studies have explored integrating LLMs with robotic control systems to allow robots to perform language-based tasks. For example, approaches such as SayCan (Ahn et al., 2022), Grounded Decoding (GD) (Huang et al., 2023), Translated Large Models (Huang et al., 2022a), and Inner Monologue (Huang et al., 2022b) use LLMs to generate action sequences and map them to robot skills. Yet, many existing approaches fail to generate structured and executable sub-task sequences from high-level instructions, especially in complex environments. Although recent methods based on vision-language models (VLMs) have improved the flexibility of reasoning, and some have introduced feedback mechanisms to optimize reasoning, they still rely on implicit representations and lack explicit modeling of object attributes and relationships between objects. Moreover, general-purpose VLMs are not always reliable sources of metric spatial information. Errors in object localization or spatial relation understanding may be propagated into downstream planning and robot motion if the VLM output is used directly. This limitation often leads to inconsistent or ambiguous task decomposition and results in the inefficient use of feedback mechanisms.

Therefore, robust and reliable language-driven task execution remains a pressing challenge in two key areas: First, regarding task planning, traditional methods either enumerate all possible next actions or generate free-form text that may refer to objects not present in the environment, thereby limiting their adaptability in complex real-world scenarios. Second, regarding feedback mechanisms, most studies employ overly subjective strategies that lack systematic design, and frequent interventions further reduce execution efficiency.

To address these challenges, we propose a VLM-based Planning and Feedback Embodied Agent (PFEA) that integrates structured sub-task planning with effective feedback-driven refinement. Rather than relying on a single general-purpose VLM for all perception, reasoning, and localization requirements, PFEA adopts a modular perception-planning design. Specifically, CoOR uses VLMs for semantic task decomposition and explicit reasoning over object attributes and inter-object relationships, while open-vocabulary detection with RGB-D sensing provides spatial grounding and metric object coordinates for execution. In this way, CoOR manages the limitations of VLM-based reasoning by constraining high-level plans with grounded perceptual information, instead of directly treating VLM-generated spatial descriptions as precise robot coordinates. We also introduce a Result-Oriented Feedback (ROF) mechanism to provide systematic and efficient feedback to improve execution robustness. The proposed framework unifies language understanding, visual perception, and execution feedback within a single embodied agent architecture, enabling adaptive replanning and reliable execution in both simulated and real-world environments.

In this paper, we make the following contributions:

- We develop a training-free, modular embodied agent system that harnesses the zero-shot capabilities of vision-language models. This provides a highly interpretable and efficient solution for real-world robotic control, bypassing the need for large-scale trajectory data typical of black-box end-to-end approaches.
- We propose a CoOR planner that overcomes the limitations of implicit representations and free-form text generation by explicitly modeling object attributes and inter-object relationships. This enables the generation of structured, executable sub-task sequences. Additionally, we introduce a ROF mechanism to facilitate robust closed-loop execution.
- We establish a comprehensive experimental suite for tabletop object rearrangement and manipulation, covering diverse object types, task categories such as stacking, sorting, placement, packing, and desktop organization, as well as different prompt conditions. Extensive results validate that our framework substantially outperforms existing baselines in complex multi-step manipulation tasks.

## 2 Related work

### 2.1 Planning with language models

Recent studies have explored how language models can be organized into robotic planning pipelines rather than used only as general-purpose text generators. Representative systems, including SayCan (Ahn et al., 2022) and Inner Monologue (Huang et al., 2022b), combine high-level language reasoning with low-level robotic skills to produce executable sub-task sequences (Singh et al., 2022; Xu et al., 2025; Dorbala et al., 2023; Wang et al., 2024). Subsequent work further improves this hierarchical paradigm through code generation, structured planning, and skill selection, making language-based planners more applicable to embodied task execution (Huang et al., 2024; Huynh and Nguyen, 2025; Mon-Williams et al., 2025; Liang et al., 2023; Ma X. et al., 2024; Lee et al., 2025; Rana et al., 2023; Morin et al., 2025).

However, these methods often struggle in dynamic environments where the state of objects may change over time. The challenges are particularly severe for tasks with strict sequential dependencies, as errors in early steps can propagate and lead to task failure. In addition, the lack of explicit grounding in environmental information may cause the generated plans to be inconsistent with the actual scene. In PFEA, we address these limitations by incorporating object attribute information from the environment via CoOR, enabling the planner to generate ordered and context-aware sub-tasks at each planning step, thereby improving robustness and execution reliability.

### 2.2 Task planning feedback

As the number of LLM-based studies continues to grow, many works have begun to incorporate closed-loop feedback mechanisms to improve task execution success rates (Renze and Guven, 2024; Madaan et al., 2023; Li and Zhao, 2025; Hsing, 2025). By integrating feedback into the planning-execution loop, these approaches enable agents to iteratively refine their action sequences based on environmental observations and execution outcomes (Li et al., 2025; Ahmadi et al., 2026). Specifically, Inner Monologue (Huang et al., 2022b) proposed both active and passive feedback mechanisms for language-based embodied systems, allowing the agent to adjust its behavior during execution. DEPS (Wang et al., 2023) further introduced self-explanations that provide feedback upon failure during planning, thereby improving the correction of initial plans generated by large language models (Mon-Williams et al., 2025; Menon et al., 2025; Shinn et al., 2023). These methods demonstrate that feedback plays a crucial role in enhancing the robustness and adaptability of LLM-based planning systems (Bhat et al., 2024; Li and Zhong, 2025).

However, the above feedback designs may rely on subjective or heuristic evaluations, lack a unified assessment framework, or introduce redundant high-level feedback queries that disrupt execution and reduce efficiency. This does not mean that intermediate feedback is unnecessary; for highly dynamic or safety-critical tasks, intermediate feedback remains essential. In the relatively structured tabletop manipulation tasks considered in this work, PFEA adopts a ROF mechanism with a dedicated task evaluator that focuses on goal-level execution outcomes. By providing a more objective and concise evaluation signal, ROF enables adaptive replanning when the final task goal is not achieved, thereby reducing redundant high-level interventions while remaining compatible with low-level execution monitoring and safety checks.

### 2.3 Vision-language embodied agents

As LLM-based agents continue to advance, the field of embodied intelligence has seen rapid development (Singh et al., 2025). The core objective is to enable close collaboration between LLMs and external tools, allowing LLMs not only to understand and generate language but also to control physical robots to perform tasks (Shridhar et al., 2022; Reed et al., 2022; Brohan et al., 2023; Wang et al., 2025). In this way, these models are essentially given *hands* and *feet*, shifting from being able to *speak* to being able to *act*.

Embodied control with large models can be broadly categorized according to system architecture into end-to-end embodied control (Brohan et al., 2022) and agent-based hierarchical control (Vemprala et al., 2024). Comprehensive surveys can be found in Ma Y. et al. (2024) and Shao et al. (2025). End-to-end methods train models on datasets containing language, vision, and robotic actions (Zitkovich et al., 2023; Wen et al., 2025; Kim et al., 2024). More recently, vision-language-action (VLA) models such as $\pi_{0.5}$ and $\pi_{0.6}^{*}$ have demonstrated the potential of generalist robot policies for long-horizon manipulation, online adaptation, and complex task execution in dynamic real-world environments (Intelligence et al., 2025b; a). These advances indicate that end-to-end VLA models are becoming increasingly capable of integrating high-level planning and low-level control within a unified policy. However, such models typically require substantial robot data and computational resources, and their internal decision processes may remain difficult to interpret, constrain, or systematically correct after execution failures (Cai et al., 2024).

In contrast, agent-based hierarchical control decomposes embodied intelligence into perception, planning, feedback, and action execution modules. General multi-agent frameworks such as MetaGPT have further demonstrated how role-based decomposition and collaboration can support complex task solving (Hong et al., 2023). This architecture is cost-effective because it can reuse existing LLMs/VLMs without costly end-to-end retraining (Liu et al., 2024; Hu et al., 2023). More importantly, in the context of HAI, hierarchical architectures provide transparency and interactive intelligence. By decoupling high-level semantic reasoning from low-level physical execution, the system can generate transparent intermediate plans, allowing human users to monitor and understand the agent’s decision-making process, which is fundamental to building human-robot trust. Such modularity also enables the agent to adapt its high-level logic based on environmental feedback, making it suitable for diverse and loosely specified human-centered environments.

Therefore, this paper focuses on embodied control through agent-based systems that utilize LLMs and VLMs. In this context, PFEA is complementary to recent VLA models rather than a replacement for them. While VLA models provide powerful end-to-end perception-action policies, PFEA contributes an external planning and feedback layer with explicit object-level reasoning, grounded metric perception, interpretable sub-task generation, and result-oriented replanning. These properties allow future VLA policies to be integrated into PFEA as action executors or low-level control modules, while PFEA preserves transparent task management and systematic failure recovery.

## 3 PFEA system architecture

### 3.1 Problem formulation

This work investigates the problem of enabling an embodied agent to interpret high-level natural language instructions in a visual environment, executing them by decomposing the task into a sequence of sub-tasks, while ensuring robust task completion through a closed-loop feedback mechanism. To formalize this problem, we define the task representation, the state representation, the planning and execution process, and the feedback mechanism.

#### 3.1.1 Task representation

A high-level task is represented as T∈L , where L is the space of natural language instructions provided by the human user (e.g., “Stack the letters in alphabetical order”).

#### 3.1.2 Observation representation

At any time step t , the environmental observation is defined as $S_{t}$ , which corresponds to the current RGB image captured by the agent. Let $O_{t}^{n}$ be the set of n objects detected from $S_{t}$ in the scene.

#### 3.1.3 Planning and execution structure

The Planner generates a sequence of k sub-tasks based on the task T and the current observation $S_{t}$ : $p=\left(s_{1},s_{2},\ldots ,s_{k}\right)$ , where each $s_{j}\in L$ is a semantic step (e.g., “Place G on A″”). The converter C maps each $s_{j}$ to an executable primitive action $a_{j}\in A$ where A is the set of all possible actions, such that the full execution sequence is $A=\left(a_{1},a_{2},\ldots ,a_{k}\right)$ .

#### 3.1.4 Feedback update rules

After executing A , the evaluator E assesses the final state $S_{t+k}$ with respect to the task T :

$$f=E\left(T,S_{t+k}\right)\in \left\{0,1\right\},$$ (1)

where f=1 indicates successful task completion. If f=0 , the system triggers a re-planning phase: $S_{t}\leftarrow S_{t+k}$ , and generates a new plan $p^{\prime }$ until f=1 or a maximum retry limit is reached.

Algorithm 1 PFEA framework execution process.

**Input:** Instruction T∈L , RGB-D image I , Max retries M

**Output:** Success flag f∈{0,1}

Initialize r←0 , f←0 , t←0 ;
**while** f=0 ***and*** r<M **do**
 
${\left\{\phi \left(o_{i}\right)\right\}}_{i=1}^{n}\leftarrow \left[color_{i},shape_{i},size_{i},co_{i},r_{i}\right]$

 
$p\leftarrow {\text{VLM}}_{\text{CoOR}}\left(T,S_{t},{\left\{\phi \left(o_{i}\right)\right\}}_{i=1}^{n}\right)$

 **foreach** $s_{j}\in p$ **do**
  
$a_{j}\leftarrow C\left(s_{j}\right)$ ▹ Map to primitive action
Execute $a_{j}$

 
$S_{t+k}=\left(I_{t+k},O_{t+k}\right)$ ▹ Observe post-execution state
 
$f\leftarrow E\left(T,S_{t+k}\right)$

 **if** f=0 **then**
  
r←r+1

  
$S_{t}\leftarrow S_{t+k}$

**return** f

### 3.2 PFEA overview

**FIGURE 1:**
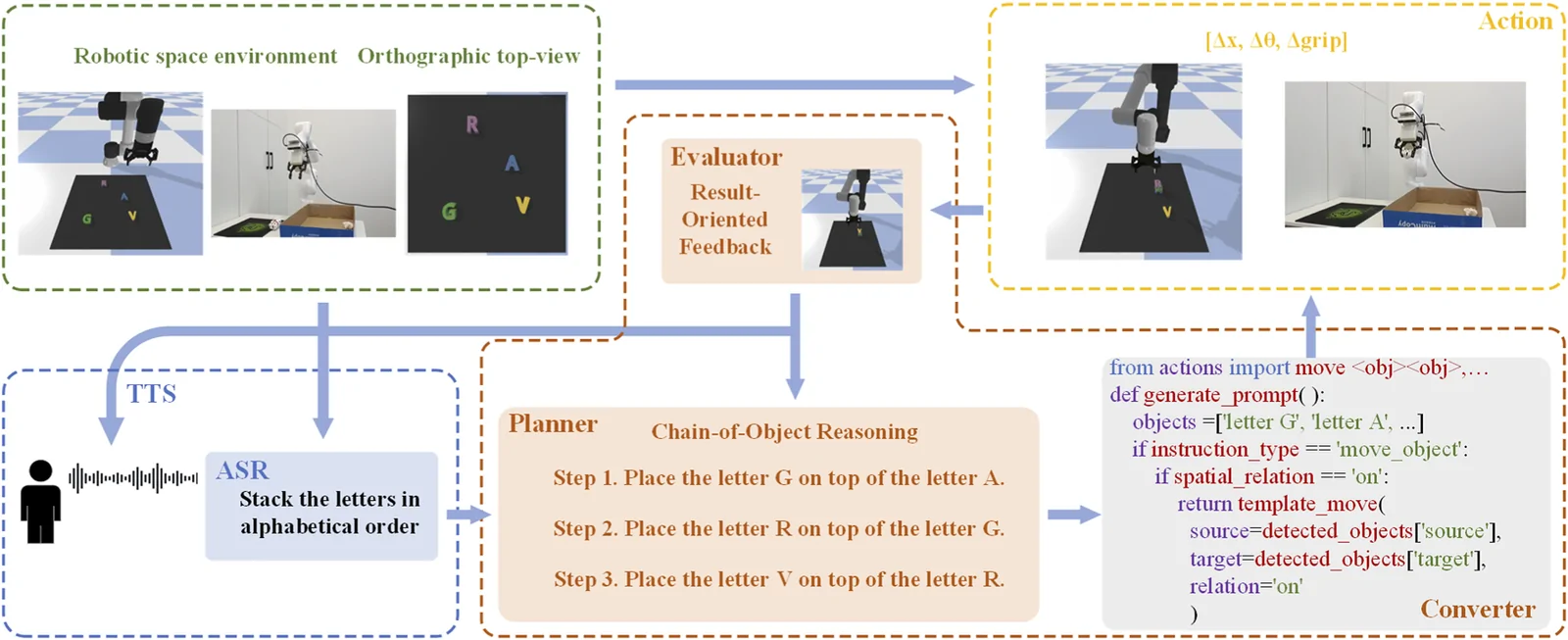
PFEA system architecture. The PFEA agent processes human voice commands via speech recognition, vision-language planning, code conversion, robotic action execution, and task assessment, and produces a final natural language response.

In this section, we describe the individual components and implementation details of PFEA, as illustrated in Figure 1 and described in Algorithm 1.

#### 3.2.1 Speech information processing

To facilitate direct interaction with humans, our embodied agent is equipped with capabilities analogous to human *hearing* and *speech*. This enables natural spoken interaction between users and the robot. The embodied agent receives human speech input, which is first processed by an Automatic Speech Recognition (ASR) model (Wu et al., 2023) to convert the audio signal into a textual instruction T∈L . This instruction is then passed to the agent’s planner for high-level language-based planning and execution, effectively enabling the robot to perceive spoken input.

The text output of PFEA is synthesized using a Text-To-Speech (TTS) model (Jiang et al., 2023), allowing the robot to converse in a human-like manner. For ASR and TTS, we adopt models trained within the FunAudioLLM framework (An et al., 2024): SenseVoice for multidimensional voice analysis and CosyVoice for highly natural speech synthesis.

#### 3.2.2 Vision-language agent

This is the core of our designed embodied intelligence system, which functions as the robot’s *brain*. The agent is composed of three parts: the planner, the converter, and the evaluator.

##### 3.2.2.1 Planner

Upon receiving high-level instructions T∈L from the user and the current RGB observation $S_{t}$ , our agent employs CoOR, a VLM-based planning mechanism that interprets abstract instructions and decomposes them into executable sub-tasks $p=\left(s_{1},s_{2},\ldots ,s_{k}\right)$ . Unlike conventional LLM-only approaches, CoOR enables explicit reasoning about object attributes (e.g., corner counts, color, shape, size) and spatial relationships, automatically inferring the execution order without explicit coordinate specifications. The planner does not require task-specific model fine-tuning. Instead, it uses a structured CoOR prompt that asks the VLM to first identify task-relevant objects and attributes, then determine their ordering or relational constraints, and finally output a concise list of executable sub-tasks in a fixed format. To reduce randomness and improve reproducibility, we use deterministic decoding settings for planning and code conversion in all experiments. The system primarily utilizes GLM-4V (GLM et al., 2024) as the VLM, because it provides strong multimodal reasoning, supports local/open-source deployment, and allows a consistent model family to be used across the planner, converter, evaluator, and baseline implementations. We also conducted interface-level and preliminary prompt tests with Qwen3-VL (Bai et al., 2025), GPT-4V (Yang et al., 2023; Ouyang et al., 2022), Gemini (Team et al., 2023), and other models, which confirmed that the CoOR prompt format can be transferred to different VLM backbones.

##### 3.2.2.2 Converter

Given the planner output, each semantic sub-task $s_{j}$ must be translated into executable robotic control commands. We introduce a converter module C powered by LLMs, which maps linguistic instructions to low-level control primitives: $a_{j}\leftarrow C\left(s_{j}\right)$ , where $a_{j}$ denotes an executable action in the skill library. The converter employs structured programmatic prompts that guide the LLM to parse spatial relationships (e.g., “put X into Y″”) and generate corresponding Python code. Each action $a_{j}$ is executed sequentially by the robot, with temporal coordination ensuring proper sequencing of grasping, lifting, and placing operations. This language-to-code translation bridges semantic reasoning and low-level motor control, enabling the robot to perform complex spatial manipulation tasks directly from natural language instructions.

##### 3.2.2.3 Evaluator

Upon executing the complete action sequence $A=\left(a_{1},a_{2},\ldots ,a_{k}\right)$ , the system invokes the ROF mechanism, where a VLM-based evaluator E assesses task completion based on the final visual state $S_{t+k}$ . The evaluator outputs a binary decision signal as shown in Equation 1, which is fed back to the planner for replanning. When f=0 , the updated state $S_{t+k}$ triggers replanning with $p^{\prime }$ , enabling iterative refinement until task success is achieved or a maximum retry threshold is reached. This closed-loop feedback mechanism ensures robust execution under real-world uncertainties.

#### 3.2.3 Robotic grasping and action execution

We adopt the open-vocabulary detection model CLIP (Radford et al., 2021; Minderer et al., 2023) to construct a framework that maps semantic labels to spatial coordinates. The semantic labels from the converter module are cross-modally matched with real-time RGB-D visual features to localize target objects. Using aligned depth measurements and calibrated camera intrinsics, we reproject the 2D image pixel coordinates into the 3D camera coordinate frame. These 3D camera coordinates are then transformed to world coordinates through hand-eye calibration, achieving precise mapping between image and physical spaces. During execution, the RGB-D camera continuously captures real-time frames while the detector tracks target objects. The system employs visual servoing to iteratively adjust joint positions based on pixel-level errors, ensuring robust alignment despite detection noise or mechanical inaccuracies. Once aligned, the system uses inverse kinematics to compute optimal joint configurations, and the manipulator executes joint-space trajectories to reach the target position accurately.

Algorithm 1 summarizes the overall execution process of the proposed PFEA framework. The PFEA takes as inputs a high-level natural language instruction T∈L and the current scene observation $S_{t}$ . At each iteration, the system first extracts object attributes from the current observation, then invokes the VLM planner to generate an executable task sequence $p=\left(s_{1},s_{2},\ldots ,s_{k}\right)$ . Each step $s_{j}$ is then converted by the Converter C into an executable action $a_{j}\in A$ , and the robotic arm executes the resulting action sequence $A=\left(a_{1},a_{2},\ldots ,a_{k}\right)$ . After execution, the system observes the updated environment $S_{t+k}$ and evaluates task completion using the evaluator $E\left(T,S_{t+k}\right)$ . If the task is not completed (f=0) , the updated state is fed back into the planner to generate a revised plan $p^{\prime }$ , enabling closed-loop task execution.

## 4 Vision-language planning and evaluation

In this section, we first detail the proposed CoOR as a concrete implementation of the planner p for structured sub-task generation. After that, we present the feedback mechanism E i.e., ROF, for improving execution robustness.

### 4.1 Chain-of-Objects Reasoning

We introduce CoOR, a mechanism that enables vision-language models to explicitly reason about object attributes and execution order for sequential manipulation tasks. CoOR addresses the challenge of decomposing high-level instructions involving multiple objects into executable robot manipulation primitives without explicit coordinate specifications. Given a task T and observation $S_{t}$ , CoOR operates through the following three interconnected stages.

#### 4.1.1 Attribute extraction and object grounding

The vision-language planner first identifies all task-relevant objects and extracts their attributes. Formally, for each detected object $o_{i}\in O_{t}^{n}$ , we construct an attribute vector (Equation 2):

$$\phi \left(o_{i}\right)=\left[color_{i},shape_{i},size_{i},co_{i},r_{i}\right]$$ (2)

where $co_{i}$ represents geometric properties (e.g., number of corners) and $r_{i}$ denotes the spatial relationship. $color_{i},shape_{i},size_{i}$ denote object attributes used for reasoning. For instance, the instruction “Stack the objects from most to fewest corners” is explicitly inferred as:

$$o_{1}=\text{green star},\;co_{1}=5,$$

$$o_{2}=\text{yellow hexagon},\;co_{2}=6,$$

$$o_{3}=\text{blue quadrilateral},\;co_{3}=4,$$

$$o_{4}=\text{purple triangle},\;co_{4}=3.$$

#### 4.1.2 Sequential ordering via chain reasoning

CoOR introduces a *reasoning chain* that establishes the execution order based on the task semantics and object attributes. We define an ordering function (Equation 3):

$$\sigma :\;O_{t}^{n}\times T\to \left\{1,2,\ldots ,n\right\},$$ (3)

which maps objects to positions in the execution sequence. For stacking tasks with ordering constraint C (e.g., “most to fewest”), σ is computed as (Equation 4):

$$\sigma \left(o_{i}\right)=\text{rank}\left(co_{i},C\right),$$ (4)

where rank(⋅) is a ranking function that scores target candidates based on their geometric attributes from $co_{i}$ .

#### 4.1.3 Spatial relationship grounding

Each semantic step $s_{j}\in p$ encodes spatial relationships that determine target locations. We define a relationship mapper (Equation 5):

$$R\left(s_{j}\right)=\left(o_{\text{source}},o_{\text{target}},r\right),$$ (5)

where $o_{\text{source}}$ denotes the object to be manipulated and grasped, $o_{\text{target}}$ denotes the reference object indicating the target destination, and r∈{“on”,“into”,“beside”} specifies the spatial relationship. For instance, for $s_{j}=\text{“Place green star on yellow hexagon”}$ , we have:

-
$o_{\text{source}}=\text{green star}$ (to be grasped)
-
$o_{\text{target}}=\text{yellow hexagon}$ (destination reference)
-
r=“on” (vertical stacking relationship)

Where r represents the spatial constraints specified in the instruction T , enabling the VLM to disambiguate relational semantics without explicit coordinate supervision. Unlike geometric object attributes, r is dynamically grounded in the semantic context of the instruction and task intent. For example, instructions such as stack, insert, or align induce different relational priors.

The VLM generates the action sequence using CoOR prompting (Equation 6):

$$p={\text{VLM}}_{\text{CoOR}}\left(T,S_{t},{\left\{\phi \left(o_{i}\right)\right\}}_{i=1}^{n}\right).$$ (6)

The resulting plan p is structured as a reasoning chain that sequentially captures object identification, attribute-based ordering, and relationally grounded action generation. As demonstrated in Table 1, CoOR enables the model to decompose high-level instructions into a series of executable steps by jointly reasoning over object attributes and their explicit spatial relationships.

**TABLE 1:** Example output of CoOR prompting for an object-stacking instruction.

Example output
Instruction: “Stack objects from most to fewest corners.”
Reasoning Chain
1. Identify: green star (5), yellow hexagon (6), blue quad (4)
purple triangle (3)
2. Order: hexagon (6) → star (5) → quad (4) → triangle (3)
3. Spatial Relationship
$s_{1}$ : (star, hexagon, on)
$s_{2}$ : (quad, star, on)
$s_{3}$ : (triangle, quad, on)
4. Generate: [“Place star on hexagon”
”Place quad on star”
”Place triangle on quad”]

### 4.2 Result-oriented feedback

The ROF mechanism is designed to use the final environmental state after task execution as the criterion for judging task success, rather than relying on subjective evaluations of intermediate processes. This mechanism is triggered after the completion of a planned sub-task sequence, performing a holistic scene assessment through a vision-language model and outputting structured control decisions. After all sub-actions derived from the high-level instruction are executed, the system captures the current scene using a camera. Unlike methods that repeatedly invoke high-level VLM/LLM feedback after intermediate steps, ROF performs a goal-level semantic evaluation after the planned sequence has been executed. This design reduces redundant high-level feedback calls and computational overhead in the relatively structured tabletop manipulation tasks considered in this work. Importantly, ROF is not intended to replace low-level execution monitoring or safety checks. Failures such as unsuccessful grasps, unreachable poses, or abnormal controller states can still be detected by the execution module and used to stop.

The feedback prompt template can be adapted for different task types (e.g., stacking tasks, placement tasks, sorting tasks), while the core evaluation logic remains unchanged. Ultimately, the evaluator decides whether the task has been successfully completed and whether the replanning mechanism should be triggered. The evaluator E uses a feedback control prompt template, such as the following for placement tasks, which contains two observation dimensions:1) The spatial aggregation judgment that Evaluates whether objects are spatially grouped, and detects any scattered objects, and 2) the container placement judgment that evaluates whether all target objects have been placed into designated containers.

## 5 Experiments

Our research employs a two-stage experimental design. We first conduct simulation experiments to verify whether PFEA can interpret high-level natural language commands, decompose them into a series of steps, and successfully perform tasks such as desktop organization (Section 5.1). The primary advantage of simulation is its ability to provide controllable environmental variables and high reproducibility, which enable reliable evaluation of the system’s effectiveness and generalization under precisely defined conditions. This is difficult to achieve on physical hardware, where complex and uncontrollable real-world factors hinder precise validation of the system’s core mechanisms.

Subsequently, real-world experiments assess the applicability of PFEA in practical scenarios, building on the simulation results (Section 5.3). Physical hardware environments introduce real-world interactions and environmental disturbances that cannot be fully replicated in simulations. This enables validation of the system’s adaptability under complex real-world conditions. Consequently, this phase provides insights unattainable through simulation alone, specifically determining the practical efficacy of PFEA in performing tasks in genuine human environments. This serves as a complement and provides final validation of the simulation findings.

### 5.1 Simulation robotic manipulation

#### 5.1.1 Simulation setup

**FIGURE 2:**
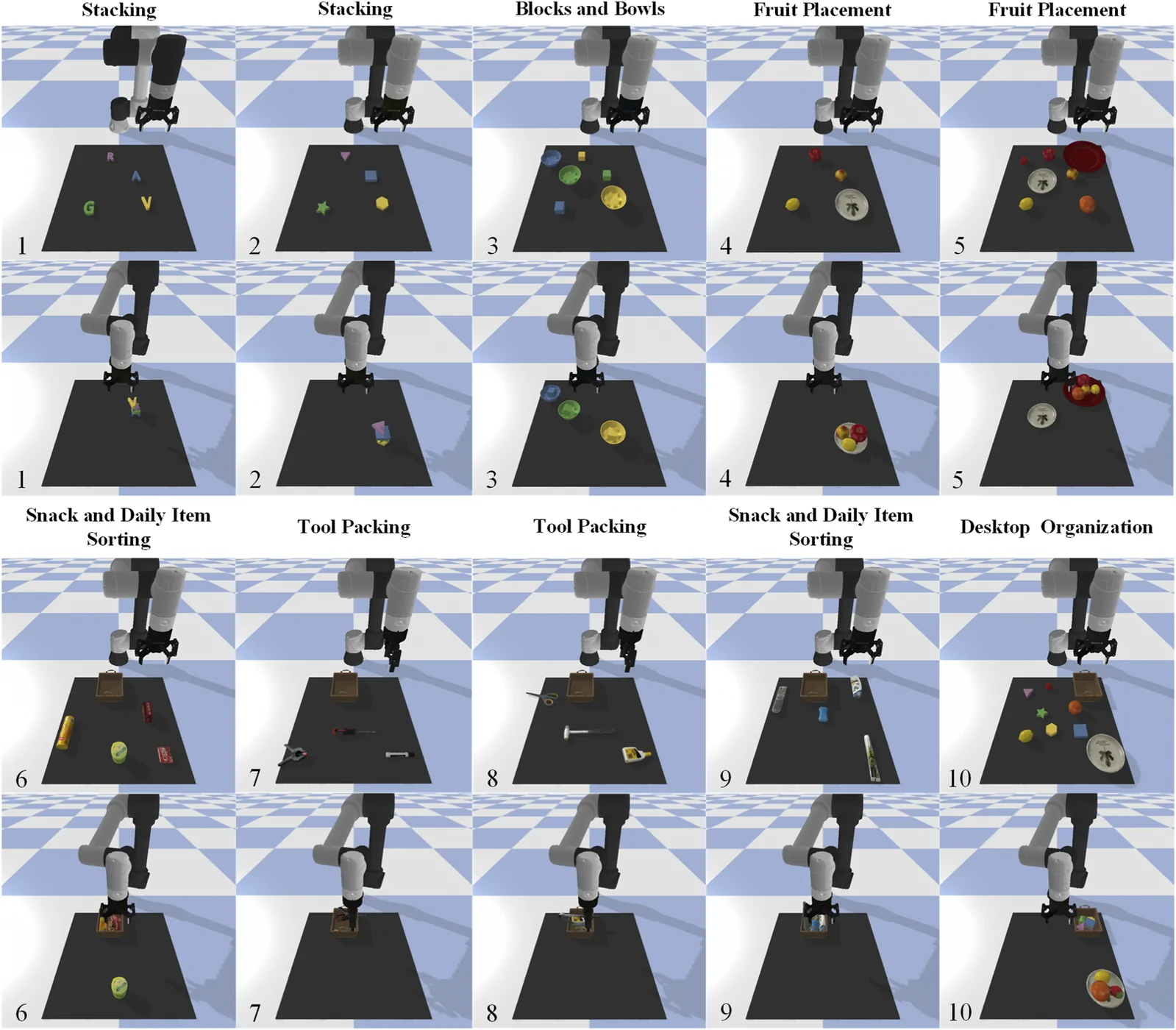
Simulation scenarios and task outcomes. Initial desktop configurations (top row) and corresponding post-execution states (bottom row) for ten distinct tasks. Each column represents a specific task category as labeled, with detailed metrics provided in Table 2.

We conduct experiments in a simulated tabletop manipulation environment based on RAVENS (Zeng et al., 2021). The simulated setup includes a UR5e robotic arm, a Robotiq 2F-85 gripper, an Intel RealSense D435i RGB-D camera, and various tabletop objects, as illustrated in Figure 2. Using these 10 environments, we developed a custom task suite comprising twenty high-level natural language instruction tasks. The 10 tasks include task-planning prompts, while the remaining ten are unprompted. The agent was not trained on any of these twenty tasks. Each task is tested twenty times. The tasks are categorized as follows:

- Stacking: Stacking letters or blocks according to alphabetical order or corner count (“Stack the blocks in descending order of the number of corners”).
- Blocks and Bowls: Rearranging blocks into color-matching bowls (“Put each block into the bowl of the same color”).
- Fruit Placement: Placing all fruits into a designated dish (“Place all fruits into the red plate”).
- Snack and Daily Item Sorting: Storing snacks and daily-use items into boxes (“Put all snacks except the chewing gum into the box”).
- Tool Packing: Sorting and packing tools into appropriate containers (“Pack the tools into the box”).
- Desktop Organization (Mixed Category): Grouping different types of objects into separate containers to achieve a tidy desktop (“Put all fruits in the bowl and all blocks in the box”).

Each of these tasks presents unique challenges. For instance, in stacking tasks, the formation of composite structures in early stages can degrade object representations. The 3D overlaps among letter-shaped blocks often lead to confusion in texture features, increasing the likelihood of task failure. To address this, the agent schedules less interfering objects later in the stacking sequence. Similarly, in fruit placement tasks, placing a large red apple first into a small bowl may block or displace other fruits due to spatial constraints. These examples highlight the critical role of stepwise planning in task success, underscoring the importance of the agent planner’s ability to prioritize execution order.

We use three metrics to evaluate task performance: success rate (SR), executability (Exe), and feedback-adjusted success ratio (FAS). SR is the proportion of trials in which all task objectives are successfully achieved. Exe is the proportion of actions in the generated plan that are both feasible in the given environment and relevant to the current task. FAS is the proportion of successful cases where feedback is used to correct errors. SR and FAS are derived from predefined rules in the simulator for simulation experiments, while they are obtained through human observation on real robots. Exe is determined by human analysis of the planner’s generated plans.

#### 5.1.2 High-level task execution

Figure 2 presents the visual results of completed tasks. Experimental results show that the agent can reliably perform language understanding, task planning, conversion, execution, and evaluation, completing the assigned high-level tasks and providing voice feedback upon success. The success rate for each task is presented in Table 2. The performance of PFEA was evaluated across various experimental scenarios and tasks. The first ten tasks are prompt-based, while the latter ten are non-prompt tasks (unseen tasks). Experimental scenarios 1 through 10 correspond to the ten environments shown in Figure 2, arranged from left to right and top to bottom.

**TABLE 2:** PFEA task performance across twenty seen and unseen tasks.

Scene	Type of task	Tasks	PFEA (SR)
1	Stacking	Stack the letter blocks in alphabetical order	90%
2	Stacking	Stack objects in descending order based on the number of corners	80%
3	Blocks and Bowls	Place each block into the bowl that matches its color	90%
4	Fruit Placement	Place the fruits onto the plate	85%
5	Fruit Placement	Place all the fruits into the red plate	90%
6	Snack and Daily Item	Put all the snacks into the box, excluding the chewing gum	70%
7	Tool Packing	Put all the tools into the box	80%
8	Tool Packing	Organize the tools on the desk	80%
9	Snack and Daily Item	Put all the daily-use items into the box	75%
10	Mixed Category	Place the fruits onto the plate and store the blocks in the box	80%
Seen Tasks Total			82%
1	Blocks and Bowls	Put the geometrically symmetric letters into the box	35%
2	Stacking	Stack the objects in ascending order based on the number of sides	55%
3	Blocks and Bowls	Place three blocks into bowls with a non-matching color	75%
5	Fruit Placement	Place some of the fruits into the white plate and the rest into the red plate	90%
8	Tool Packing	Organize the tools on the desk (replace a claw hammer)	70%
9	Snack and Daily Item	Put all items on the desk into the box	80%
10	Blocks and Bowls	Place the block with the most sides into the box	85%
10	Mixed Category	Place the fruits into the box and the blocks onto the plate	75%
10	Fruit Placement	Place only the fruits into the box	90%
10	Stacking	Stack all the blocks sequentially onto the plate	85%
Unseen Tasks Total			74%

In this experiment, we considered four object categories commonly encountered in human-centered embodied AI scenarios: fruits, snacks, daily-use items, and tools. High-level natural language instructions are given to the agent, such as “Place all the fruits into the red bowl” or “Organize the tools on the desk”. Upon receiving the instruction, the agent first processes it through its planner module, while simultaneously acquiring RGB images from an onboard camera to support planning. The agent then sequentially executes each planned sub-task. Through its converter and execution modules, the agent identifies task-relevant target points and maps positions from image coordinates to real-world coordinates. The robotic arm is then actuated to carry out the planned manipulation actions. After execution, the agent invokes its evaluator to determine whether the task has been completed. If successful, the agent awaits the next instruction. If not, the planner is re-invoked to revise the plan based on the current task state.

#### 5.1.3 Unseen high-level task execution

We next present experiments on unseen tasks. These experiments serve two main purposes: First, to verify whether the VLM-driven agent can autonomously infer and execute the correct action sequence without relying on any step-by-step task prompts. Second, we aim to evaluate the system’s generalization capability with minimal task-specific guidance. In these experiments, we vary only the input high-level natural language commands while keeping all other components of the agent unchanged. The commands were deliberately designed to differ from the scenarios explicitly embedded in the base prompt template, preventing the agent from relying on superficial similarities in prompts to accomplish the tasks.

Therefore, we designed the following generalization experiments: In stacking tasks, the experiment entirely altered the expected stacking order, requiring the agent to automatically replan the execution sequence from scratch. In the blocks and bowls task, color matching was intentionally removed, compelling the agent to form new object-to-container associations. In fruit placement tasks, instructions were modified to require fruits to be distributed across two plates instead of one. In the snack and daily item sorting and tool packing tasks, we introduced previously unseen items that were never mentioned in any prompt to test the agent’s ability to plan and execute tasks involving entirely novel objects. In the desktop organization task, the usual object-to-container mappings were reversed (e.g., fruits were placed in boxes, and blocks were placed in bowls). In a particularly challenging setup, we combine multiple task types without any prior hint. For example, we require the agent to perform block stacking inside a plate as part of the desktop organization task. Experimental results show that the proposed agent performs strongly on unseen tasks. This strong performance underscores PFEA’s significant advantage in zero-shot generalization. Detailed success rates are presented in Table 2.

### 5.2 Baseline comparison

To further validate the effectiveness of our proposed agent architecture, we conducted comparative experiments. We employ three types of experimental baselines. The first baseline directly connects GLM-4V (GLM et al., 2024) to the robotic controller without CoOR, ROF, or the converter module. In this setting, the high-level semantic instruction and RGB observation are given to GLM-4V, whose output is directly passed to the execution module, while the execution module provides object localization based on visual matching and RGB-D sensing. The second baseline, Grounded Decoding (GD) (Huang et al., 2023), integrates an LLM with an affordance model to bias generated actions toward objects and skills that are feasible in the current scene. The third baseline, Inner Monologue (Huang et al., 2022b), extends language-based planning with environmental feedback, allowing the model to update subsequent decisions according to intermediate observations and execution results. Our work focuses on embodied task execution rather than improving the LLM itself. Therefore, to ensure a fair comparison, both our framework and the baseline methods employ the same underlying LLM family. Specifically, we use the open-source GLM-4 as the language model and GLM-4V as the vision-language model (GLM et al., 2024). This choice is also necessary for implementing GD and Inner Monologue, because these methods require access to LLM output-token probabilities for grounded action selection, whereas the OpenAI API currently does not provide the required token probabilities in our experimental setting. For the affordance models in GD and Inner Monologue, we follow Huang et al. (2023) and adopt the open-vocabulary detector OWL-ViT (Minderer et al., 2023; 2022). All baselines share the same RGB-D observations, object sets, robot skill library, and execution interface as PFEA; the differences lie only in the planning, grounding, conversion, and feedback mechanisms under evaluation.

Overall, the simulation results show that PFEA substantially outperforms the baselines in average success rate, as detailed in Table 3. To determine whether this improvement is consistent across tasks rather than driven by a few easy cases, we further analyze the task-level distribution of success rates in Table 4. For seen tasks, PFEA achieves the highest mean success rate and the smallest standard deviation, with success rates ranging from 70% to 90%. In contrast, the baselines show lower means and larger task-level variation. In particular, GD ranges from 25% to 75%, indicating greater sensitivity to task type.

**TABLE 3:** Average success rates of PFEA and the three baselines in the simulation environment.

	Seen tasks			Unseen tasks		
Methods	Exe	FAS	SR	Exe	FAS	SR
GLM-4V (GLM et al., 2024)	65%	-	38%	34%	-	31%
GD (Huang et al., 2023)	70%	-	51%	65%	-	42%
Inner Monologue (Huang et al., 2022b)	72%	23%	63%	63%	29%	50%
PFEA	**90%**	**25%**	**82%**	**83%**	**35%**	**74%**

Bold values indicate the best performance.

**TABLE 4:** Distribution of task-level success rates across different methods in the simulation environment.

	Seen tasks			Unseen tasks		
Methods	Mean	Std	Min-max	Mean	Std	Min-max
GLM-4V (GLM et al., 2024)	38.0%	10.6%	20%–50%	28.5%	13.1%	10%–50%
GD (Huang et al., 2023)	51.0%	14.7%	25%–75%	42.0%	16.5%	10%–60%
Inner Monologue (Huang et al., 2022b)	64.0%	13.9%	45%–85%	47.5%	17.2%	15%–65%
PFEA	**82.0%**	**6.7%**	**70%–90%**	**74.0%**	17.3%	**35%–90%**

Bold values indicate the best performance.

For unseen tasks, all methods exhibit larger variability, reflecting the difficulty of generalizing to novel instructions and object configurations. PFEA still maintains the highest mean success rate of 74.0% and the highest lower bound among the four methods, although its standard deviation increases to 17.3% and its success rates range from 35% to 90%. The lower end of this range is mainly associated with abstract reasoning tasks, such as identifying geometrically symmetric letters, where all methods experience reduced performance. These results indicate that PFEA improves not only overall success rate but also task-level robustness, particularly in seen tasks, while retaining a clear advantage under unseen task conditions.

### 5.3 Real-world robotic manipulation

#### 5.3.1 Experiment setup

In the real-world experiments, we employed a JAKA Mini 2 robotic arm and an Intel RealSense D435i RGB-D camera. We design four tabletop manipulation tasks covering both high-level and low-level natural language instructions. An egocentric camera mounted on the robotic arm captures RGB images at 640 × 480 resolution.

We implemented four challenging tasks (Table 5). All tasks are performed without task-specific training. In the task of placing a metal clip into a cardboard box, we deliberately omit the clip’s color (e.g., “pink metal clip”) to ensure the agent relies on multimodal perception of physical properties rather than shortcut cues based on color. The third and fourth tasks are high-level instructions that do not specify concrete objects, requiring ordered planning and execution in open-ended scenarios.

**TABLE 5:** Results of PFEA in four real-world tasks.

Tasks	PFEA (SR)
Put the iron clip into the box	80%
Put the stapler in the box	65%
Put everything on the table in the box	70%
Everything but the stapler goes in the box	60%
Total	68%

#### 5.3.2 Real-world task execution

**FIGURE 3:**
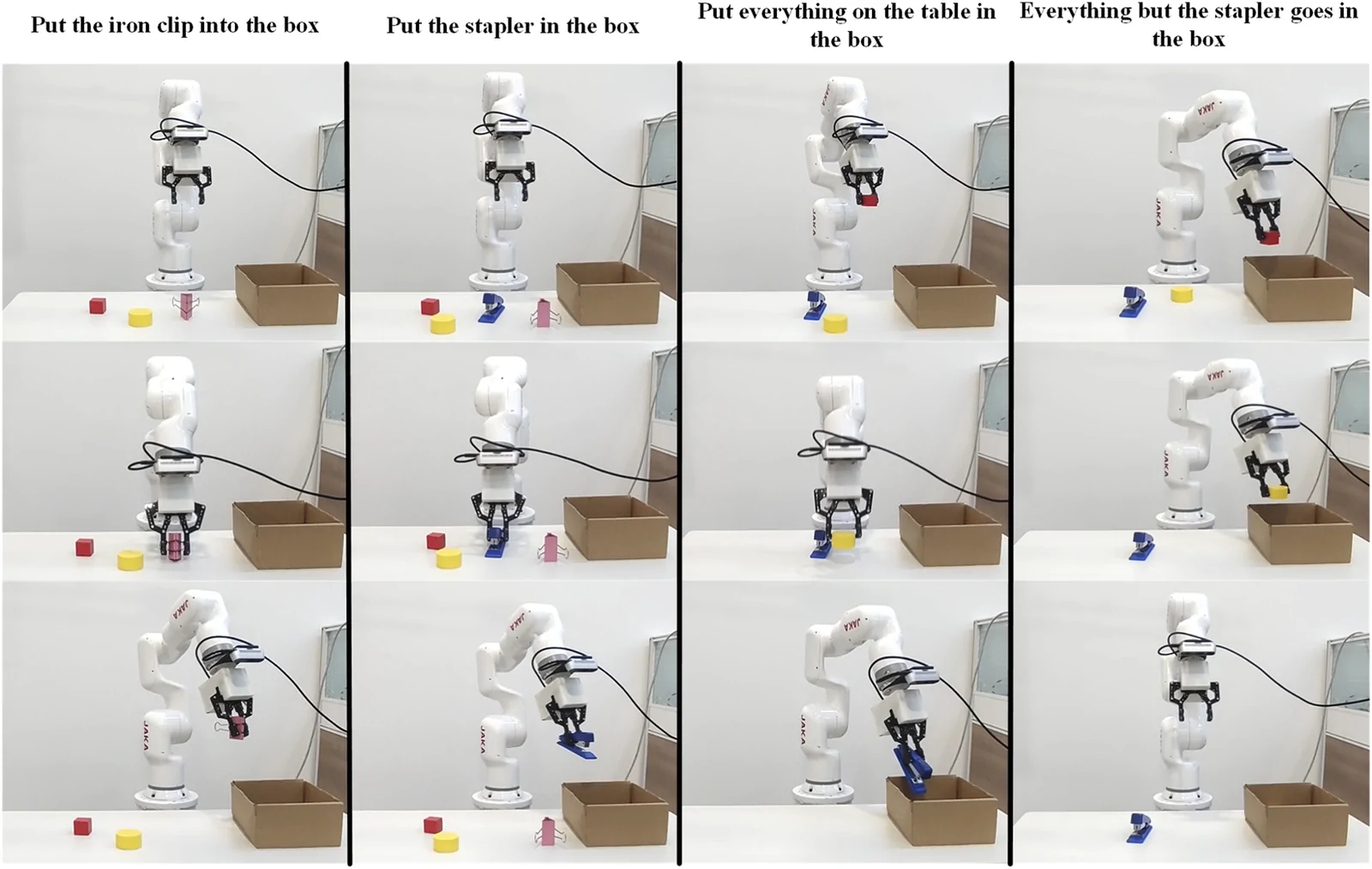
Real-world task execution across four sequential task demonstrations: Each column shows a complete task sequence from the initial state to completion, corresponding to the experimental tasks detailed in Table 5.

The agent shows strong performance in real-world tasks, as illustrated in Figure 3. Across four real-world task scenarios, our agent achieved a 16% higher success rate than baseline approaches. This improvement stems from effective task planning and feedback mechanisms. Detailed experimental results are presented in Tables 5, 6. Each task was tested twenty times. Real-world experiments confirm the effectiveness of the agent in practical applications. Across diverse testing environments and real-world settings, the agent demonstrates strong adaptability, highlighting the feasibility and advantages of the framework for human-centered tasks in real-world deployments.

**TABLE 6:** Average success rates of PFEA and the three baselines in the real-world.

	Real-world tasks		
Methods	Exe	FAS	SR
GLM-4V (GLM et al., 2024)	60%	-	30%
GD (Huang et al., 2023)	68%	-	44%
Inner Monologue (Huang et al., 2022b)	70%	20%	52%
PFEA	**86%**	**27%**	**68%**

Bold values indicate the best performance.

### 5.4 Ablation study and observational findings

#### 5.4.1 Ablation study

In the ablation study, we consider two settings: removing the planner and removing the evaluator. The tasks are identical to those in Table 2, comprising twenty tasks. Unlike the comparative experiments, we modify the success rate calculation. In the comparative setting, each trial is scored as binary (1 for success, 0 for failure). In contrast, the ablation study adopts a graded scheme: partial execution is assigned 0.25, full task completion is scored as 1, and failure as 0. Results are averaged over twenty trials per task and then across all tasks. The results are reported in Table 7 (w/o = without).

**TABLE 7:** Comparative experiments across twenty tasks.

	Average success rate		
Tasks	PFEAw/o planner	PFEAw/o evaluator	PFEA
Seen Tasks	62%	66%	**82%**
Unseen Tasks	45%	53%	**74%**

Bold values indicate the best performance.

#### 5.4.2 Observational findings

Hierarchical methods exhibit limitations in low-level execution. Experimental observations indicate that the discrepancy between planning and execution success rates primarily stems from the following factors: minor inaccuracies in position recognition, deviations in the two-finger gripper, and friction between the gripper and the object. Notably, in real-world scenarios, friction significantly affects grasping. In addition, the grasping angle introduces further variability. For instance, when grasping a metal clip, the success rate differs depending on whether the front or rear end is grasped. Finally, we observe that for systems without a feedback mechanism, initial execution success is critical, as such systems lack the ability to detect and correct errors once a plan is generated. This makes the overall performance highly sensitive to the quality of the initial action planning and execution. In contrast, methods equipped with feedback can iteratively refine their actions based on observations, thereby improving robustness to early errors.

Qualitative observations from the recorded videos indicate that tasks using the feedback mechanism generally require longer completion time than those without feedback. This is expected because ROF introduces additional evaluation, replanning, and corrective execution steps when execution errors or unsatisfactory intermediate outcomes are detected. These results suggest a robustness-efficiency trade-off: although feedback may increase execution time, it enables the agent to recover from errors and improves the likelihood of successful task completion. Since the main objective of this work is to improve the reliability and task completion capability of the proposed agent in complex tabletop manipulation scenarios driven by high-level natural language instructions, our evaluation primarily focuses on task success rate rather than minimizing completion time.

## 6 Conclusion

In this paper, we propose PFEA, a vision-language embodied agent designed for real-world robotic tasks. The proposed framework enables robots to interpret and execute high-level natural language instructions in human-centered environments. We first introduce a speech recognition and synthesis module that facilitates bidirectional conversion between human speech and text, enabling natural human–robot interaction. We then develop a task planning agent with visual perception capabilities. By leveraging vision-language models (VLMs), the planner decomposes high-level instructions into a sequence of executable actions. In addition, an agent converter is designed to translate natural language instructions into executable Python control code. An agent evaluator is further incorporated to provide assessment and feedback, which plays a critical role in completing complex tasks. Finally, an action execution module is implemented on both simulated and real robotic platforms. Experimental results demonstrate that the proposed framework effectively enables robots to perform human-centered tasks driven by high-level natural language instructions in both simulated and real-world environments. In addition, feedback-enabled trials tend to take longer than trials without feedback, mainly due to the extra evaluation, replanning, and corrective execution procedures.

Beyond the specific performance gains observed in this study, PFEA also suggests several design principles for future LLM and VLM-based embodied planning systems. First, high-level foundation-model reasoning should be coupled with explicit perceptual grounding, so that generated plans remain constrained by the objects, attributes, and spatial relationships present in the current environment. Second, embodied agents should separate semantic task decomposition from metric localization and low-level control, allowing improvements in future foundation models or action policies to be incorporated without redesigning the entire framework. Third, result-oriented feedback remains important even as stronger models emerge, because physical execution errors, perception uncertainty, and environmental changes cannot be fully eliminated by language or vision-language reasoning alone. These principles indicate that the main value of PFEA is not limited to a snapshot of performance with a particular model, but lies in its modular closed-loop design for grounding, planning, execution, and feedback. Nevertheless, PFEA still has limitations. The generalizability of low-level control policies restricts the range of tasks that can be executed reliably, and the limited interpretability of VLMs may reduce planning stability. In addition, ROF is best suited to tasks where the environment remains relatively stable during a planned sub-task sequence. For highly dynamic or safety-critical scenarios, ROF should be combined with event-triggered intermediate feedback and real-time safety monitoring so that the robot can abort or adapt before completing an unsuccessful action sequence. Future work will investigate how newer foundation models, general-purpose action models, and richer multimodal feedback can be integrated into this closed-loop framework to support more robust and transferable embodied agents.

## Data Availability

The original contributions presented in the study are included in the article/Supplementary Material, further inquiries can be directed to the corresponding author.

## References

[B1] Ahmadi I. Taji M. Kashani A. M. Jadidi A. Kashani S. Khalaj B. (2026). Mallvi: a multi agent framework for integrated generalized robotics manipulation. arXiv preprint arXiv:2602, 16898.

[B2] Ahn M. Brohan A. Brown N. Chebotar Y. Cortes O. David B. (2022). Do as i can, not as i say: grounding language in robotic affordances. arXiv preprint arXiv:2204.01691.

[B3] An K. Chen Q. Deng C. Du Z. Gao C. Gao Z. (2024). Funaudiollm: voice understanding and generation foundation models for natural interaction between humans and llms. arXiv preprint arXiv:2407.04051.

[B4] Bai S. Cai Y. Chen R. Chen K. Chen X. Cheng Z. (2025). Qwen3-vl technical report. arXiv preprint arXiv:2511.21631.

[B5] Bhat V. Kaypak A. U. Krishnamurthy P. Karri R. Khorrami F. (2024). Grounding llms for robot task planning using closed-loop state feedback. arXiv preprint arXiv:2402, 08546.

[B6] Brohan A. Brown N. Carbajal J. Chebotar Y. Dabis J. Finn C. (2022). Rt-1: robotics transformer for real-world control at scale. arXiv preprint arXiv:2212.06817.

[B7] Brohan A. Brown N. Carbajal J. Chebotar Y. Chen X. Choromanski K. (2023). Rt-2: vision-language-action models transfer web knowledge to robotics control. arXiv preprint arXiv:2307.15818.

[B8] Brown T. Mann B. Ryder N. Subbiah M. Kaplan J. D. Dhariwal P. (2020). Language models are few-shot learners. Adv. Neural Information Processing Systems 33, 1877–1901. 10.48550/arXiv.2005.14165

[B9] Cai W. Ponomarenko I. Yuan J. Li X. Yang W. Dong H. (2024). Spatialbot: precise spatial understanding with vision language models. arXiv preprint arXiv:2406.13642.

[B10] Capel T. Brereton M. (2023). “What is human-centered about human-centered ai? a map of the research landscape,” in Proceedings of the 2023 CHI Conference on Human Factors in Computing Systems, 1–23.

[B11] Dorbala V. S. Mullen J. F. Manocha D. (2023). Can an embodied agent find your “cat-shaped mug”? Llm-based zero-shot object navigation. IEEE Robotics Automation Lett. 9, 4083–4090. 10.1109/lra.2023.3346800

[B12] Glm T. Zeng A. Xu B. Wang B. Zhang C. Yin D. (2024). Chatglm: a family of large language models from glm-130b to glm-4 all tools. arXiv preprint arXiv:2406.12793.

[B13] Hong S. Zheng X. Chen J. Cheng Y. Wang J. Zhang C. (2023). Metagpt: meta programming for multi-agent collaborative framework. arXiv preprint arXiv:2308.00352 3.

[B14] Hsing N. (2025). Mirror: modular internal processing for personalized safety in llm dialogue. arXiv preprint arXiv:2506.00430.

[B15] Hu Y. Lin F. Zhang T. Yi L. Gao Y. (2023). Look before you leap: unveiling the power of gpt-4v in robotic vision-language planning. arXiv preprint arXiv:2311, 17842.

[B16] Huang W. Abbeel P. Pathak D. Mordatch I. (2022a). “Language models as zero-shot planners: extracting actionable knowledge for embodied agents,” in *International Conference on Machine Learning* (PMLR), 9118–9147.

[B17] Huang W. Xia F. Xiao T. Chan H. Liang J. Florence P. (2022b). Inner monologue: embodied reasoning through planning with language models. arXiv preprint arXiv:2207.05608.

[B18] Huang W. Xia F. Shah D. Driess D. Zeng A. Lu Y. (2023). Grounded decoding: guiding text generation with grounded models for embodied agents. Adv. Neural Inf. Process. Syst. 36, 59636–59661. 10.48550/arXiv.2303.00855

[B19] Huang W. Wang C. Li Y. Zhang R. Fei-Fei L. (2024). Rekep: spatio-temporal reasoning of relational keypoint constraints for robotic manipulation. arXiv preprint arXiv:2409.01652.

[B20] Huynh T. N. Nguyen K.-D. (2025). Integrative ai framework for robotics: Llm-enabled reinforcement learning in object manipulation and task planning. Robotics Aut. Syst. 195, 105197. 10.1016/j.robot.2025.105197

[B21] Intelligence P. Amin A. Aniceto R. Balakrishna A. Black K. Conley K. (2025a). π 0.6: a vla that learns from experience. arXiv preprint arXiv:2511.14759.

[B22] Intelligence P. Black K. Brown N. Darpinian J. Dhabalia K. Driess D. (2025b). π0.5: a vision-language-action model with open-world generalization. arXiv preprint arXiv:2504.16054.

[B23] Jiang Z. Liu J. Ren Y. He J. Ye Z. Ji S. (2023). Mega-tts 2: boosting prompting mechanisms for zero-shot speech synthesis. arXiv preprint arXiv:2307, 07218.

[B24] Kim M. J. Pertsch K. Karamcheti S. Xiao T. Balakrishna A. Nair S. (2024). Openvla: an open-source vision-language-action model. arXiv preprint arXiv:2406.09246.

[B25] Lee J. Lee H. Kim J. Lee K. Kim E. (2025). “Self-corrective task planning by inverse prompting with large language models,” in 2025 IEEE International Conference on Robotics and Automation (ICRA) (IEEE), 11017–11023.

[B26] Li B. Zhao C. (2025). Self-reflection enhances large language models towards substantial academic response. Artif. Intell. 1, 42. 10.1038/s44387-025-00045-3

[B27] Li Y. Zhong V. (2025). How well can llms provide planning feedback in grounded environments? arXiv preprint arXiv:2509.09790.

[B28] Li J. Yang L. Wei Z. Zhou H. Fang K. (2025). “Multi-agent collaborative closed-loop planning-evaluation-assessment framework: optimization and interpretability evaluation,” in 2025 4th International Conference on Intelligent Mechanical and Human-Computer Interaction Technology (IHCIT) (IEEE), 107–112.

[B29] Liang J. Huang W. Xia F. Xu P. Hausman K. Ichter B. (2023). “Code as policies: language model programs for embodied control,” in 2023 IEEE International Conference on Robotics and Automation (ICRA) (IEEE), 9493–9500.

[B30] Liu P. Orru Y. Vakil J. Paxton C. Shafiullah N. M. M. Pinto L. (2024). Ok-robot: what really matters in integrating open-knowledge models for robotics. arXiv preprint arXiv:2401, 12202.

[B31] Ma X. Patidar S. Haughton I. James S. (2024). “Hierarchical diffusion policy for kinematics-aware multi-task robotic manipulation,” in Proceedings of the IEEE/CVF Conference on Computer Vision and Pattern Recognition, 18081–18090.

[B32] Ma Y. Song Z. Zhuang Y. Hao J. King I. (2024). A survey on vision-language-action models for embodied ai. arXiv preprint arXiv:2405.14093. 10.1109/TNNLS.2025.3650584 42048203

[B33] Madaan A. Tandon N. Gupta P. Hallinan S. Gao L. Wiegreffe S. (2023). Self-refine: iterative refinement with self-feedback. Adv. Neural Information Processing Systems 36, 46534–46594. 10.48550/arXiv.2303.17651

[B34] Menon A. R. Sharma R. K. Singh P. Wang C. Ferreira A. M. Novak M. (2025). Think, act, learn: a framework for autonomous robotic agents using closed-loop large language models. arXiv preprint arXiv:2507, 19854.

[B35] Minderer M. Gritsenko A. Stone A. Neumann M. Weissenborn D. Dosovitskiy A. (2022). “Simple open-vocabulary object detection,” in European Conference on Computer Vision (Springer), 728–755.

[B36] Minderer M. Gritsenko A. Houlsby N. (2023). Scaling open-vocabulary object detection. Adv. Neural Inf. Process. Syst. 36, 72983–73007. 10.48550/arXiv.2306.09683

[B37] Mon-Williams R. Li G. Long R. Du W. Lucas C. G. (2025). Embodied large language models enable robots to complete complex tasks in unpredictable environments. Nat. Mach. Intell. 7, 592–601. 10.1038/s42256-025-01005-x 40391151 PMC12088599

[B38] Morin S. Gupta K. Sandhu M. Gauthier C. Argenziano F. Ellis K. (2025). “Object-centric agentic robot policies,” in NeurIPS 2025 Workshop on Space in Vision, Language, and Embodied AI.

[B39] Ouyang L. Wu J. Jiang X. Almeida D. Wainwright C. Mishkin P. (2022). Training language models to follow instructions with human feedback. Adv. Neural Information Processing Systems 35, 27730–27744. 10.48550/arXiv.2203.02155

[B40] Radford A. Kim J. W. Hallacy C. Ramesh A. Goh G. Agarwal S. (2021). “Learning transferable visual models from natural language supervision,” in *International Conference on Machine Learning* (PmLR), 8748–8763.

[B41] Rana K. Haviland J. Garg S. Abou-Chakra J. Reid I. Suenderhauf N. (2023). Sayplan: grounding large language models using 3d scene graphs for scalable robot task planning. arXiv preprint arXiv:2307.06135.

[B42] Reed S. Zolna K. Parisotto E. Colmenarejo S. G. Novikov A. Barth-Maron G. (2022). A generalist agent. arXiv preprint arXiv:2205.06175.

[B43] Renze M. Guven E. (2024). Self-reflection in llm agents: effects on problem-solving performance. arXiv preprint arXiv:2405.06682.

[B44] Shao R. Li W. Zhang L. Zhang R. Liu Z. Chen R. (2025). Large vlm-based vision-language-action models for robotic manipulation: a survey. arXiv preprint arXiv:2508.13073.

[B45] Shinn N. Cassano F. Gopinath A. Narasimhan K. Yao S. (2023). Reflexion: language agents with verbal reinforcement learning. Adv. Neural Information Processing Systems 36, 8634–8652. 10.48550/arXiv.2303.11366

[B46] Shridhar M. Manuelli L. Fox D. (2022). “Cliport: what and where pathways for robotic manipulation,” in Conference on Robot Learning (PMLR), 894–906.

[B47] Singh I. Blukis V. Mousavian A. Goyal A. Xu D. Tremblay J. (2022). Progprompt: generating situated robot task plans using large language models. arXiv preprint arXiv:2209.11302.

[B48] Singh H. Das R. J. Han M. Nakov P. Laptev I. (2025). “Malmm: multi-agent large language models for zero-shot robotic manipulation,” in 2025 IEEE/RSJ International Conference on Intelligent Robots and Systems (IROS) (IEEE), 20386–20393.

[B49] Team G. Anil R. Borgeaud S. Alayrac J.-B. Yu J. Soricut R. (2023). Gemini: a family of highly capable multimodal models. arXiv Preprint arXiv:2312.11805.

[B50] Vaswani A. Shazeer N. Parmar N. Uszkoreit J. Jones L. Gomez A. N. (2017). Attention is all you need. Adv. Neural Information Processing Systems 30, 5998–6008. 10.48550/arXiv.1706.03762

[B51] Vemprala S. H. Bonatti R. Bucker A. Kapoor A. (2024). Chatgpt for robotics: design principles and model abilities. IEEE Access. 12, 55682–55696. 10.1109/ACCESS.2024.3387941

[B52] Wang Z. Cai S. Chen G. Liu A. Ma X. Liang Y. (2023). Describe, explain, plan and select: interactive planning with large language models enables open-world multi-task agents. arXiv preprint arXiv:2302.01560.

[B53] Wang T. Fan J. Zheng P. (2024). An llm-based vision and language cobot navigation approach for human-centric smart manufacturing. J. Manuf. Syst. 75, 299–305. 10.1016/j.jmsy.2024.04.020

[B54] Wang C. Xia F. Yu W. Zhang T. Zhang R. Liu C. K. (2025). “Chain-of-modality: learning manipulation programs from multimodal human videos with vision-language-models,” in 2025 IEEE International Conference on Robotics and Automation (ICRA) (IEEE), 6527–6535.

[B55] Wen J. Zhu Y. Li J. Zhu M. Tang Z. Wu K. (2025). Tinyvla: towards fast, data-efficient vision-language-action models for robotic manipulation. IEEE Robotics Automation Lett. 10 (4), 3988–3995. 10.1109/LRA.2025.3544909

[B56] Wu J. Gaur Y. Chen Z. Zhou L. Zhu Y. Wang T. (2023). “On decoder-only architecture for speech-to-text and large language model integration,” in 2023 IEEE Automatic Speech Recognition and Understanding Workshop (ASRU) (IEEE), 1–8.

[B57] Xu J. Sun Q. Han Q.-L. Tang Y. (2025). When embodied ai meets industry 5.0: human-centered smart manufacturing. IEEE/CAA J. Automatica Sinica 12, 485–501. 10.1109/jas.2025.125327

[B58] Yang Z. Li L. Lin K. Wang J. Lin C.-C. Liu Z. (2023). The dawn of lmms: preliminary explorations with gpt-4v (ision). arXiv preprint arXiv:2309.17421.

[B59] Zeng A. Florence P. Tompson J. Welker S. Chien J. Attarian M. (2021). “Transporter networks: rearranging the visual world for robotic manipulation,” in *Conference on Robot Learning* (PMLR), 726–747.

[B60] Zitkovich B. Yu T. Xu S. Xu P. Xiao T. Xia F. (2023). “Rt-2: vision-language-action models transfer web knowledge to robotic control,” in Conference on Robot Learning (PMLR), 2165–2183.

